# Characterization of post‐ictal clinical signs in dogs with idiopathic epilepsy: A questionnaire‐based study

**DOI:** 10.1111/jvim.17302

**Published:** 2025-01-20

**Authors:** Aran Nagendran, Julie A. Nettifee, Dani Carter, Karen R. Muñana

**Affiliations:** ^1^ Department of Clinical Sciences North Carolina State University Raleigh North Carolina USA; ^2^ Hospital for Small Animals, Royal (Dick) School for Veterinary Studies University of Edinburgh Midlothian UK

**Keywords:** post‐ictal, quality of life, questionnaire, seizures

## Abstract

**Background:**

Post‐ictal (PI) clinical signs are a key defining stage of seizure manifestation in dogs. However, this phase remains poorly understood.

**Objectives:**

To further characterize PI signs and their relation to other parts of a seizure, and understand the owner's perception of how PI signs affect the quality of life (QOL) of the dog.

**Animals:**

Eight‐seven dogs with a diagnosis of idiopathic epilepsy from a single institution.

**Methods:**

The prospective questionnaire‐based study surveying owners of dogs previously and newly diagnosed with idiopathic epilepsy.

**Results:**

Post‐ictal signs were identified in 79/87 dogs, 5/5 of dogs with focal seizures and 74/82 of dogs with generalized seizures. Median duration of PI signs was 30 minutes (range, 5‐4320 minutes). The most common PI signs reported were disorientation (50/79) and wobbliness or clumsiness (49/79). Within a year, a change in PI signs was seen in 18/79 dogs. The administration of benzodiazepines was significantly associated with an increase in duration of PI signs (*P* = .04). Post‐ictal signs had more impact on dogs' quality of life compared with ictal signs (*P* < .01). Groupings of co‐existing PI signs identified included disorientation, blindness and deafness.

**Conclusion:**

Post‐ictal signs are a commonly reported aspect of seizures in dogs with idiopathic epilepsy, both in focal as well as generalized seizures. Co‐existence of signs could provide some valuable insight into the relevance of this particular phase of a seizure. Owner‐reported signs and documentation emphasize the need for a better understanding of PI signs in dogs with idiopathic epilepsy.

**Abbreviations**
CI: Confidence interval
EEG: Electroencephalography
PI: Post‐ictal
QOL: Quality of life
SE: Status epilepticus

## 1 INTRODUCTION

Seizures are transient, sudden, or short‐lasting events that result from excessive synchronous activity of neurons within the brain.^1^ Epilepsy, defined as ≥2 unprovoked seizures occurring at least 24 hours apart, is the most common chronic neurological problem identified in the general dog population, with an estimated prevalence of 0.62% to 0.82% in the United Kingdom.^2^, ^3^ Idiopathic epilepsy is the most frequent diagnosis in dogs with epileptic seizures; it is presumed to be genetic in origin and is diagnosed based on the exclusion of gross structural brain disease or other relevant underlying extra‐cranial causes.^4^, ^5^

Several phases are associated with seizures: a pre‐ictal or prodromal phase that precedes the epileptic seizure, an ictal phase that comprises the epileptic seizure itself, and a post‐ictal (PI) phase. The PI phase is considered a clinical abnormality of the central nervous system that arises or becomes more apparent when clinical signs of the seizure have ceased.^6^ The mean duration of PI signs in dogs with idiopathic epilepsy has been reported to range from 16 to 189 minutes after a generalized seizure and 0.9 to 33 minutes after a focal seizure, as evaluated in Dalmatians and Standard poodles, respectively.^7^ Clinical abnormalities reported in the PI period in dogs include confusion, fatigue and lethargy, hunger, thirst, and blindness.^8^, ^9^

Post‐ictal signs can appear severe in some dogs, and the presence of prolonged PI signs is an indication for initiation of anti‐seizure medication.^10^ However, the PI phase has been poorly studied in dogs. Therefore, our aim was to further characterize the PI phase in dogs with idiopathic epilepsy, including how these signs relate to the ictal and pre‐ictal phases and how relevant a part they play in contributing to quality of life (QOL).

## 2 MATERIALS AND METHODS

Medical records of dogs admitted to the Neurology service at North Carolina State University, between 2010 and 2021 were retrospectively searched to identify dogs with a diagnosis of idiopathic epilepsy with a Tier II level of confidence according to guidelines established by the International Veterinary Epilepsy Task Force (9). Owners of eligible dogs were contacted by email and asked to complete an online questionnaire. Fillable electronic questionnaires also were provided to all owners of dogs presented to the Neurology Service for evaluation of seizures between October 2021 and October 2022, and dogs with a Tier II diagnosis of idiopathic epilepsy were included in the study cohort. The questionnaire was designed for the purpose of the study and included questions about seizure semiology and history, pre‐ictal signs, ictal signs, PI signs, medication, and assessment of QO‐L (see Data S1). Owners were asked to select from a list of choices for each question, but an option of “other” associated with open‐ended answer fields was utilized to allow other observations. In relation to the PI signs, the duration of each sign in the questionnaire was categorized into 3 groups: seconds to minutes, minutes to hours, and hours to days. The frequency of these PI clinical signs was also divided into whether they occurred “always,” “sometimes,” or “never.” Owners also were asked to assess the impact of the ictal and PI phases on their dog's QOL using a Likert scale of 0 to 5, with 0 being no impact and 5 being a great impact. All owners voluntarily answered the questionnaire. Dogs were excluded if they were reported to be deceased at the time of the study or if the questionnaire was incomplete.

## 3 STATISTICS

Data was assessed for normality using a Shapiro‐Wilk Test. Demographic data was analyzed with descriptive statistics; medians and ranges were reported for data with non‐normal distribution. Fisher exact tests and Spearman correlation were performed to assess the significance of association for continuous and categorical data, respectively. Benjamini‐Hochberg correction was applied to decrease the false discovery rate in co‐existing PI signs and co‐existing ictal and PI signs. A Circos plot was devised to visualize the relationship between PI and ictal signs.^11^ All of the above tests were performed using R, version 4.1.2 (https://R-project.org/). Kornbrot rank difference test^12^ was carried out to assess the impact of the ictal and the PI phases on the QOL of the dog in question. Network analyses were conducted to assess the commonality of the presence of certain PI signs and the presence of ictal signs in relation to PI signs. Tests were performed using SAS Enterprise Guide software, Version 8.1 (SAS Institute Inc, Cary, North Carolina). A *P*‐value of <.05 was considered statistically significant.

## 4 RESULTS

A total of 108 questionnaires were received. Twenty‐one were not fully completed, which left 87 completed surveys. Of these, 38 different breeds of dogs were represented. The breeds most commonly represented in the study were the Labrador retriever (n = 15), mixed breed (n = 8), French bulldog (n = 6), golden retriever (n = 5), and beagle (n = 4). Of the 87 dogs, there were 39 females, of which 36 were neutered, and 48 males, of which 31 were neutered. The median age of onset of first seizure was 24 months (range, 6‐72 months). The median duration of seizure activity was 27 months (range, 1‐154 months). The time of day of seizure activity appeared to be non‐specific in 42/87 (48%) of dogs. Seizures occurred while the dog was at rest in 61/87 (70%) of cases. Among the 87 dogs in the study, the overall average frequency of seizure episodes (either isolated, clusters, or status epilepticus) was daily in 1 dog, weekly in 16 dogs, monthly in 42 dogs, every 2 to 6 months in 27 dogs, and yearly in 1 dog.

### 4.1 POST‐ICTAL SIGNS

**FIGURE 1:**
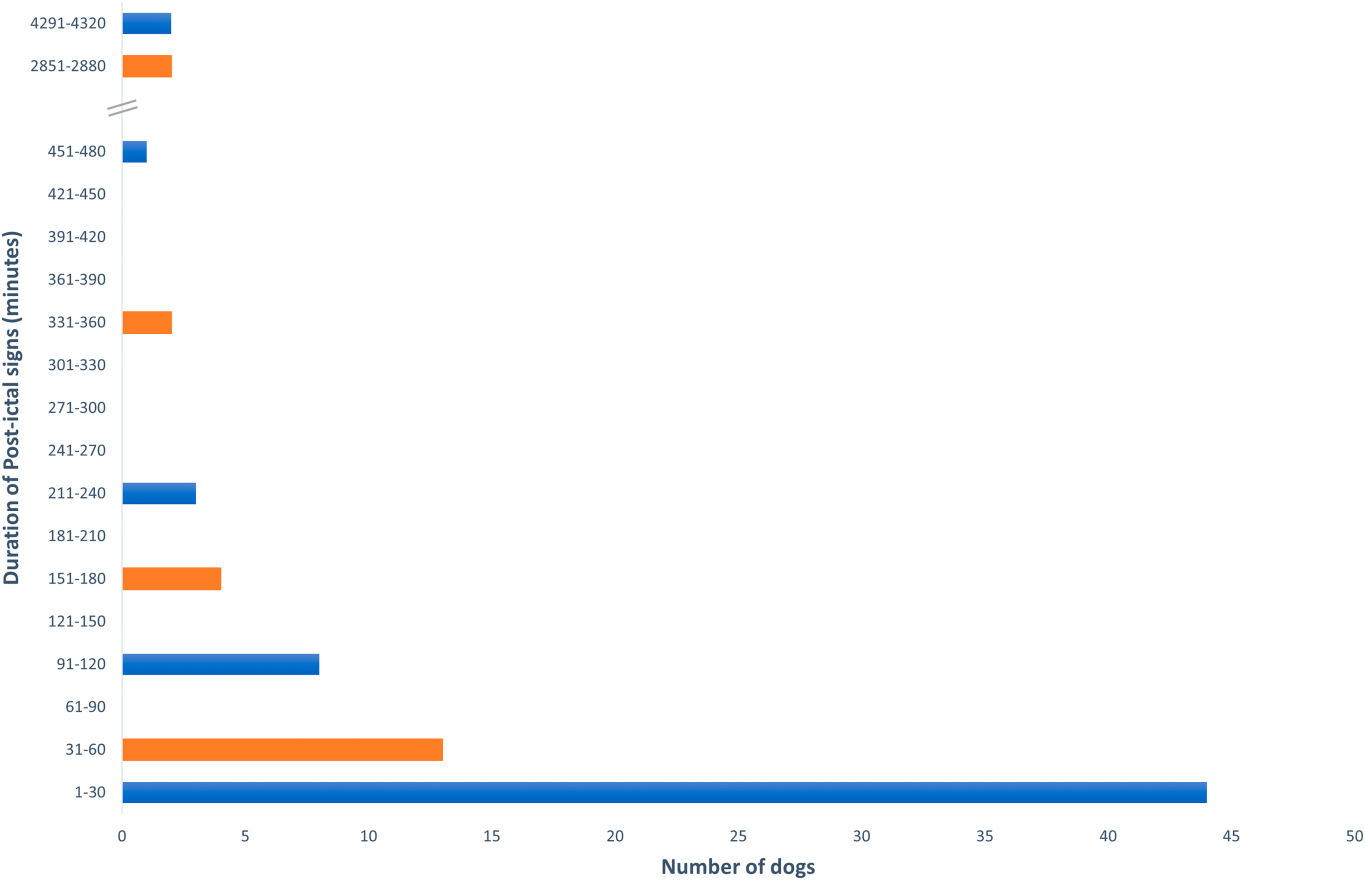
Duration of PI signs (minutes) reported by owners of dogs with idiopathic epilepsy.

**FIGURE 2:**
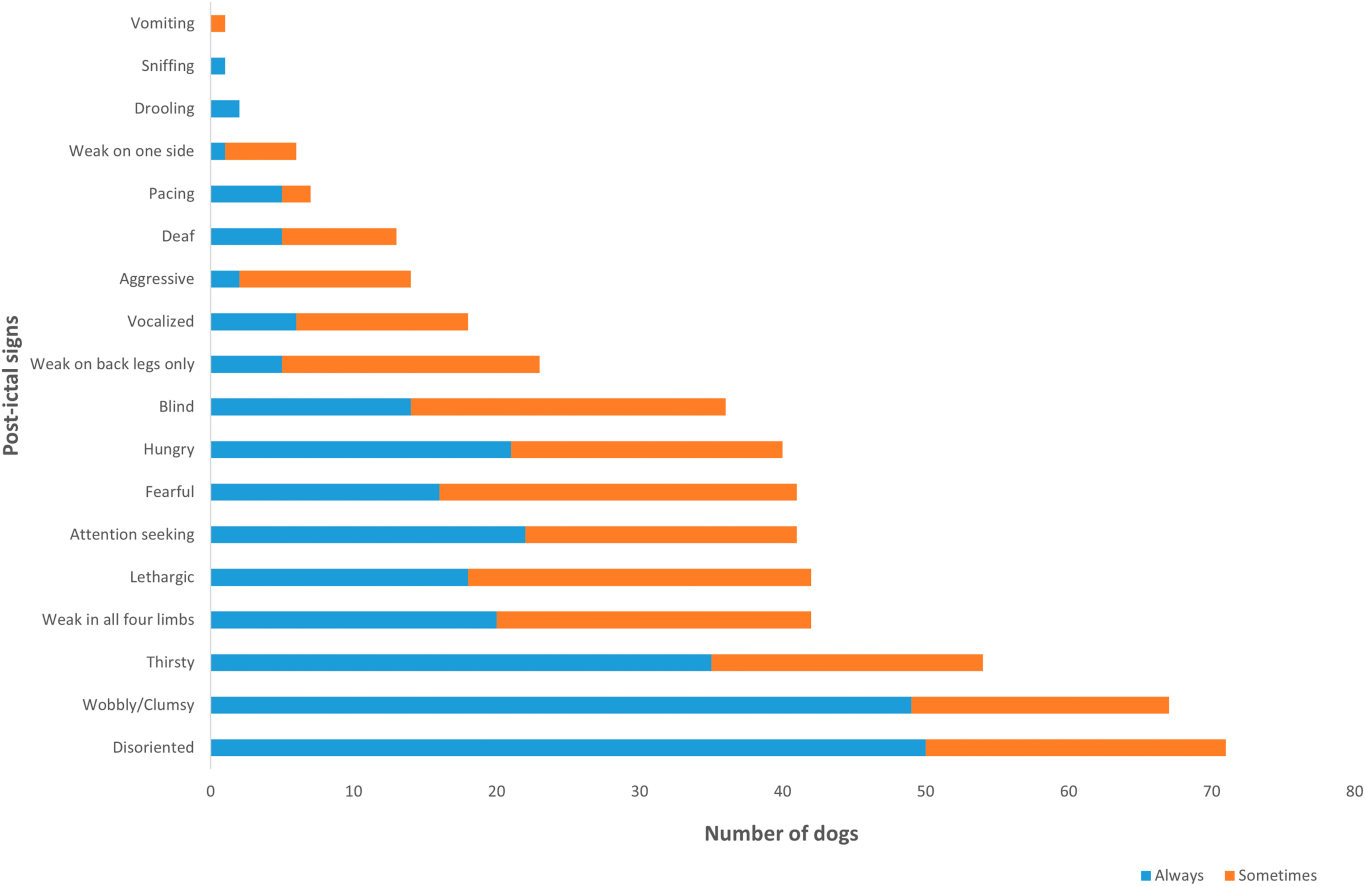
Occurrence of PI signs, reported by owners, of a total number of 79 dogs with idiopathic epilepsy. Each bar represents the number of dogs reported to have the specific PI sign; with each bar split into blue and orange representing the number of dogs that always (blue) and sometimes (orange) presented with each sign.

**FIGURE 3:**
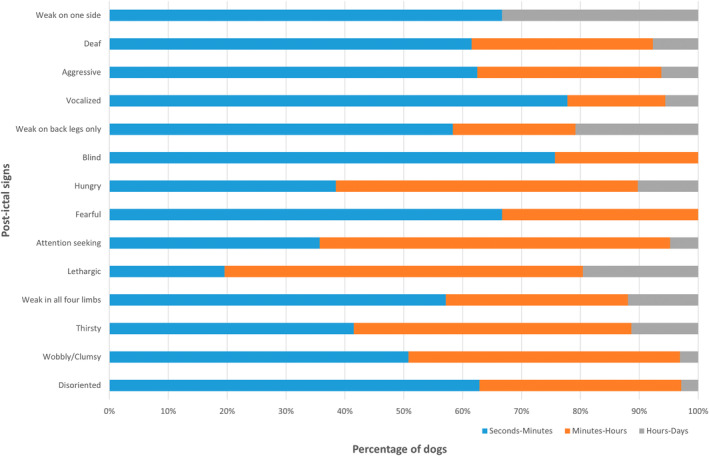
Percentage representation of duration of PI signs reported by owners in dogs with idiopathic epilepsy. One horizontal bar represents a PI sign and color segmentation represents the duration categories reported; seconds to minutes (blue), minutes‐hours (orange), and hours‐days (gray).

Post‐ictal clinical abnormalities were identified in 79/87 (90.8%; 95% confidence interval [CI], 82.7%‐95.9%) dogs. The median duration of signs was 30 minutes (range, 1‐4320 minutes); a full representation of duration is presented in Figure 1. Of the 79 dogs with PI signs, disorientation was reported in 71/79 (89.9%), wobbliness or clumsiness in 67/79 (84.8%), thirst in 54/79 (68.4%), weakness in all 4 limbs in 42/79 (53.2%), lethargy in 42/79 (53.2%), attention seeking in 41/79 (51.9%), fearfulness in 41/79 (51.9%), hunger in 40/79 (50.6%), blindness in 36/79 (45.6%), weakness affecting the rear limbs only in 23/79 (29.1%), vocalization in 18/79 (22.8%), aggression in 14/79 (17.7%), compulsive pacing in 8/79 (10.1%), weakness on 1 side (1 thoracic and 1 pelvic limb) in 6/79 (7.6%), and hypersalivation in 3/79 (3.8%). Isolated reports of sniffing and vomiting also were identified. Disorientation was the most common clinical sign to “always” be seen, with this frequency reported in 50/71 (70%; 95% CI: 59.8%‐81.1%) dogs noted to have disorientation. Fear was the most common clinical sign to only “sometimes” be seen, with this feature reported in 25/41 (61%; 95% CI: 44.5%‐75.8%) dogs noted to be fearful. The distribution of clinical signs and occurrence are depicted in Figure 2. The duration of all PI signs, apart from weakness on 1 side, varied from seconds to minutes to hours to days in the study cohort. The PI signs of vocalization and blindness were reported to last seconds to minutes in >70% of dogs that exhibited these signs; 78% and 76%, respectively. Weakness in either rear limbs or 1 side was reported to last hours to days in >20% of dogs that exhibited these signs; 33% and 22%, respectively. The distribution of the duration of clinical signs is presented in Figure 3.

### 4.2 SEIZURE SEMIOLOGY

In the study cohort, 37 dogs exhibited isolated seizures only, 10 dogs exhibited cluster seizures only and 1 dog exhibited status epilepticus. Post‐ictal signs were identified in 34/37 (91.9%) of dogs that exhibited isolated seizures only, 8/10 (80.0%) of dogs that exhibited cluster seizures only, and in the 1 dog (100%) that was reported to have status epilepticus (SE) only. The remaining dogs had a combination of seizure activity, including isolated and cluster seizures (30 dogs), cluster seizures and SE (1 dog), isolated seizures and SE (4 dogs), and isolated seizures, cluster seizures and SE (3 dogs). Of the dogs that had both clusters and isolated seizures, 28/30 (93.3%) showed PI signs. The remaining dogs that had a combination of seizure types all exhibited PI signs at least at 1 time point. Of the 28 dogs that exhibited both isolated and cluster seizures, 8 dogs experienced PI signs with both types of events, 13 only with cluster seizures, and 7 only with isolated seizures. Dogs that had a history of either SE and cluster seizures, SE and isolated seizures, or SE, cluster seizures and isolated seizures all were reported to have PI signs in all types of events.

Five dogs (6%) were reported to only have focal seizures, with the remaining 82 dogs having generalized tonic‐clonic seizures. Post‐ictal signs were seen in all (5/5) dogs presented with only focal seizure activity and 74/82 (90.2%) dogs with generalized seizures. Owners also reported the presence of suspected PI signs without witnessing an ictal event in 12/79 (15.1%; 95% CI: 8.1‐25%) dogs, which included 3/5 (60.0%) dogs that had focal seizures only and 9/74 (12.1%) dogs with a generalized seizure history. No association was identified between the duration of ictal activity and PI duration (*P* = .29).

A change in PI signs over time was identified in 18/79 (22.3%; 95% CI: 14.1%‐33.6%) dogs. The number of signs decreased in 11/18 (61.1%) dogs and increased in 7/18 (38.9%) dogs. In addition, when asked about the specific time frame of a year before questionnaire completion, a significant association was identified between an increase in PI duration and both an increase in seizure duration (Spearman correlation, *P* < .01, *r*_s_ = .38) and an increase in seizure frequency (Spearman correlation, *P* < .01, *r*_s_ = .40).

### 4.3 ANTI‐SEIZURE MEDICATION

Among the dogs with PI signs, the following anti‐seizure medications were being administered: phenobarbital (46/79; 58.2%), potassium bromide (26/79, 32.9%), zonisamide (33/79; 41.7%), levetiracetam (49/79; 62.0%), benzodiazepines (20/79; 25.3%), gabapentin (11/79; 13.9%), and pregabalin (3/79; 3.8%). Nine dogs were not receiving any medication at the time of questionnaire completion. No significant association was found between the number of anti‐seizure medications administered and PI duration (*P* = .37). However when owners were asked whether the addition of any anti‐seizure medication had an effect on PI signs, benzodiazepines were found to be significantly associated with an increase in the duration of PI signs (*P* = .04).

### 4.4 CO‐EXISTENCE OF PI SIGNS AND ICTAL SIGNS

**FIGURE 4:**
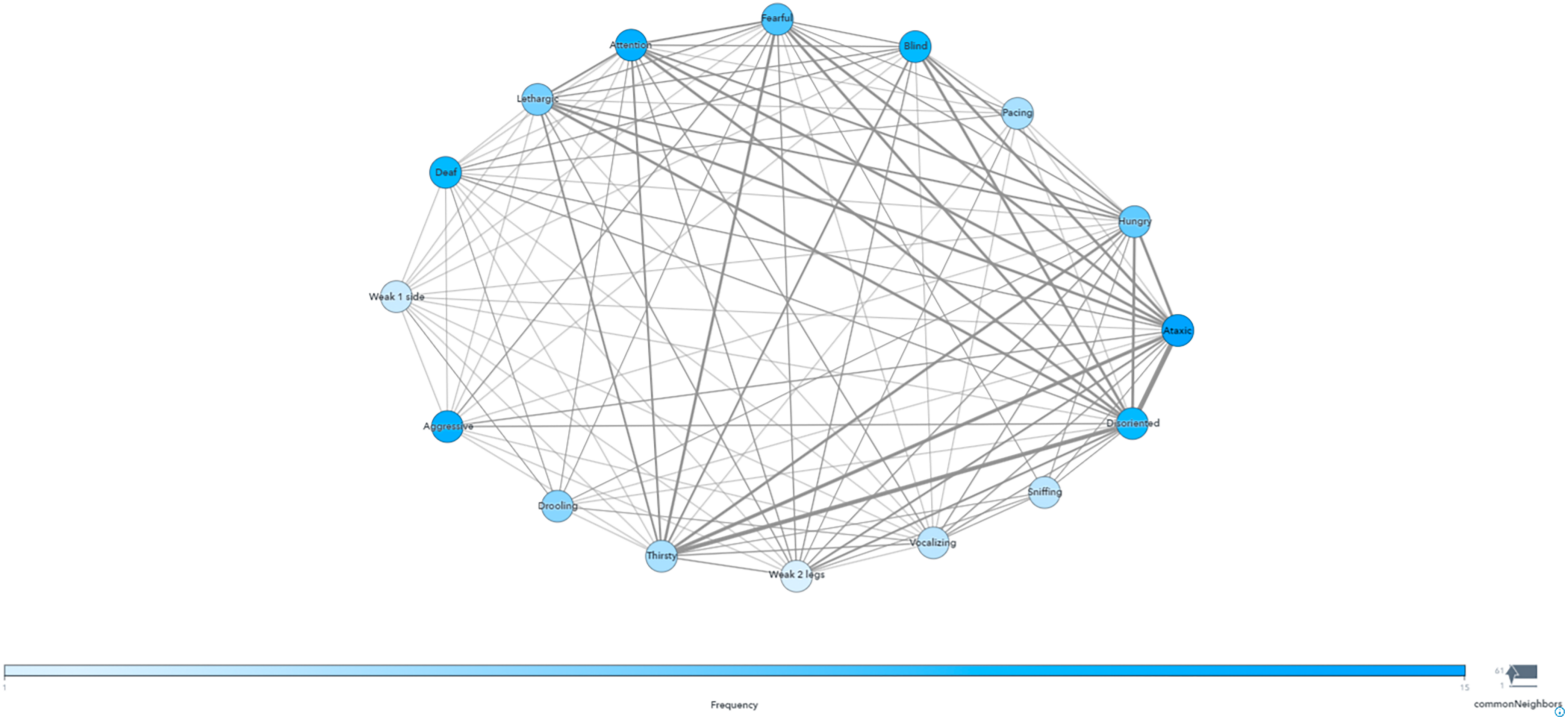
Common neighbor mapping of PI signs in our population of dogs with idiopathic epilepsy. Each PI sign is represented in the peripheral blue circles/nodes. The darker the circle (node) the more common the presenting clinical sign. Thicker interconnecting lines between the nodes indicate a stronger association.

The co‐existence of PI signs that were reported to be “always” seen was analyzed. The signs that occurred together are presented in Table 1. Fear, wobbliness, and blindness, individually, were more likely to be seen with disorientation. Hunger and wobbliness, individually, were more likely to be seen with thirst. Blindness and disorientation, individually, were more likely to be seen with deafness. Wobbliness was more likely to be seen with weakness in all 4 limbs. Multiple significant co‐existing signs included disorientation, blindness, and deafness as 1 cluster. Common neighbor mapping (Figure 4) indicated that wobbliness or clumsiness and disorientation appeared to be the common denominators in the dogs analyzed, whereas sniffing, pacing, drooling, weakness on 1 side, and vomiting appeared to be outliers. Aggression was a frequent finding but had minimal association with the other PI signs.

**TABLE 1:** The result of multiple Fisher exact tests assessing the 1‐to‐1 associative presence of PI signs in idiopathic epileptic dogs within the study group.

PI signs	Attention seeking	Fearful	Aggression	Sleepy/lethargic	Wobbly/clumsy	Blind	Disorientated	Vocalization	Weak on 4 limbs	Weak on rear limbs	Week on one side	Hungry	Thirsty	Deafness	Pacing	Salivation
Attention seeking	—	*P* = .88	*P* = 1.00	*P* = 1.00	*P* = .60	*P* = 1.00	*P* = 1.00	*P* = 1.00	*P* = 1.00	*P* = 1.00	*P* = 1.00	*P* = 1.00	*P* = 1.00	*P* = 1.00	*P* = 1.00	*P* = 1.00
Fearful	—	—	*P* = 1.00	*P* = 1.00	*P* = 1.00	*P* = .98	***P* = .01**	*P* = .63	*P* = 1.00	*P* = .55	*P* = 1.00	*P* = 1.00	*P* = 1.00	*P* = .49	*P* = 1.00	*P* = 1.00
Aggression	—	—	—	*P* = 1.00	*P* = 1.00	*P* = 1.00	*P* = 1.00	*P* = .70	*P* = 1.00	*P* = .64	*P* = 1.00	*P* = 1.00	*P* = 1.00	*P* = 1.00	*P* = 1.00	*P* = 1.00
Sleepy/lethargic	—	—	—	—	*P* = 1.00	*P* = .74	*P* = .84	*P* = .60	*P* = 1.00	*P* = 1.00	*P* = 1.00	*P* = .90	*P* = .83	*P* = 1.00	*P* = 1.00	*P* = 1.00
Wobbly/clumsy	—	—	—	—	—	*P* = .55	***P* = .01**	*P* = .87	***P* = .01**	*P* = 1.00	*P* = 1.00	*P* = .48	***P* = .02**	*P* = 1.00	*P* = 1.00	*P* = 1.00
Blind	—	—	—	—	—	—	***P* = .01**	*P* = 1.00	*P* = .56	*P* = 1.00	*P* = 1.00	*P* = .59	*P* = 1.00	***P* = .01**	*P* = 1.00	*P* = 1.00
Disorientation	—	—	—	—	—	—	—	*P* = .83	*P* = .59	*P* = 1.00	*P* = 1.00	*P* = 1.00	*P* = .67	***P* = .04**	*P* = 1.00	*P* = 1.00
Vocalization	—	—	—	—	—	—	—	—	*P* = 1.00	*P* = 1.00	*P* = 1.00	*P* = .75	*P* = .92	*P* = .10	*P* = 1.00	*P* = 1.00
Weak on 4 limbs	—	—	—	—	—	—	—	—	—	*P* = .10	*P* = 1.00	*P* = 1.00	*P* = .62	*P* = .62	*P* = 1.00	*P* = 1.00
Weak on rear limbs	—	—	—	—	—	—	—	—	—	—	*P* = 1.00	*P* = 1.00	*P* = 1.00	*P* = 1.00	*P* = 1.00	*P* = 1.00
Weak on one side	—	—	—	—	—	—	—	—	—	—	—	*P* = 1.00	*P* = 1.00	*P* = 1.00	*P* = 1.00	*P* = 1.00
Hungry	—	—	—	—	—	—	—	—	—	—	—	—	***P* = .03**	*P* = 1.00	*P* = 1.00	*P* = 1.00
Thirsty	—	—	—	—	—	—	—	—	—	—	—	—	—	*P* = 1.00	*P* = 1.00	*P* = 1.00
Deafness	—	—	—	—	—	—	—	—	—	—	—	—	—	—	*P* = 1.00	*P* = 1.00
Pacing	—	—	—	—	—	—	—	—	—	—	—	—	—	—	—	*P* = 1.00
Salivation	—	—	—	—	—	—	—	—	—	—	—	—	—	—	—	—

*Note*: The *P*‐values represented follow the Benjamini‐Hochberg correction. Significant (*P* < .05) associations are highlighted in bold.

**FIGURE 5:**
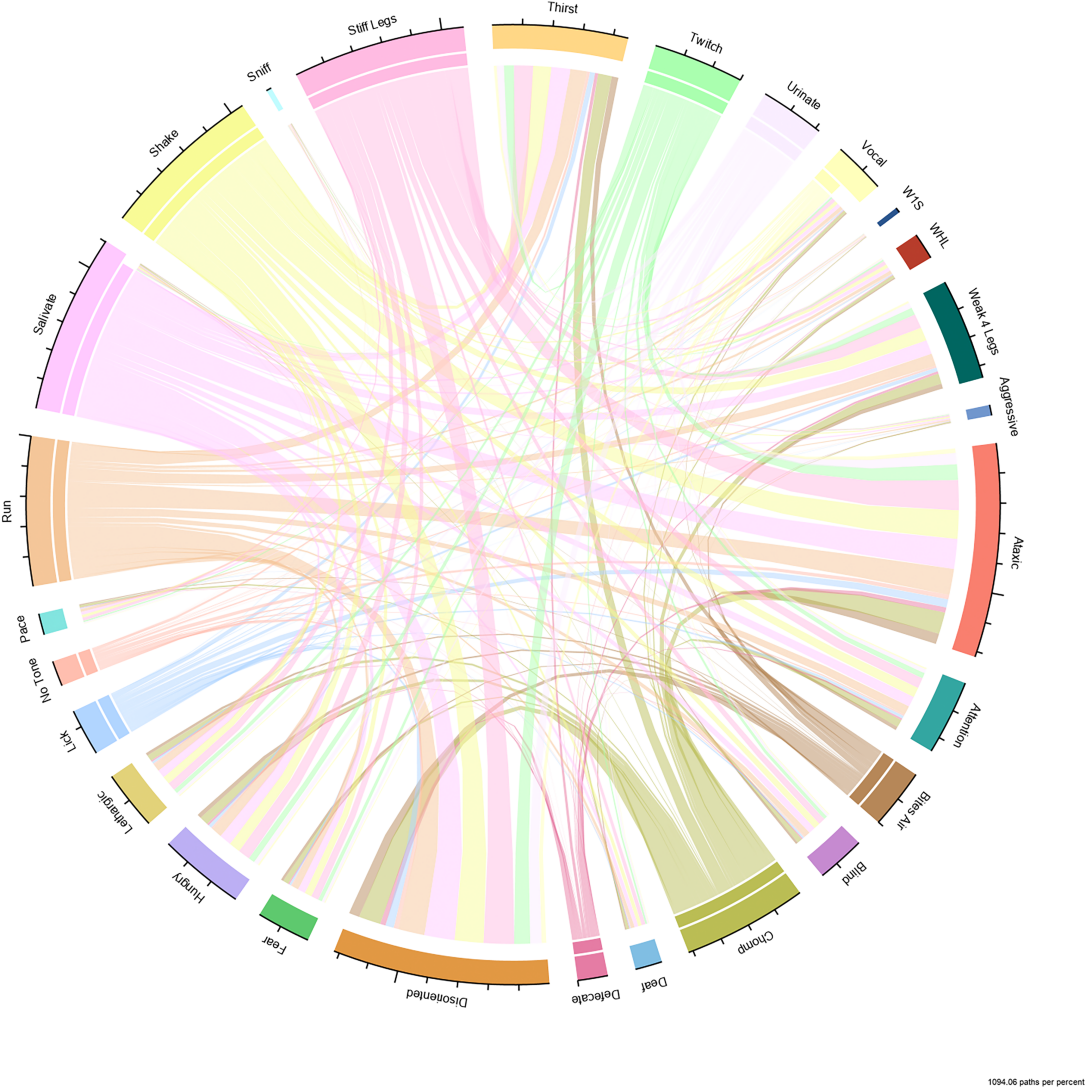
Circus plot depicting the co‐existence of ictal and the PI signs. The clinical signs with a double bar correspond to ictal signs and the ones with a single bar, to PI signs. W1S, weak 1 side; WHL, weakness in hindlimbs only. No large fusions of pathways are visualized indicating no apparent association.

The 1‐to‐1 co‐existence of ictal and PI signs also was analyzed. After Benjamini‐Hochberg correction, there were no 1‐to‐1 co‐existing ictal and PI signs with a *P*‐value <.05. A visual depiction (Circus plot) of multiple associations between ictal and PI signs is presented in Figure 5. This plot highlights that 1 subject can have many ictal and PI signs and, although no significant relationships were found between ictal and PI signs, this observation was reinforced by no evidence of strong fusions between 2 elements.

### 4.5 QUALITY OF LIFE

**FIGURE 6:**
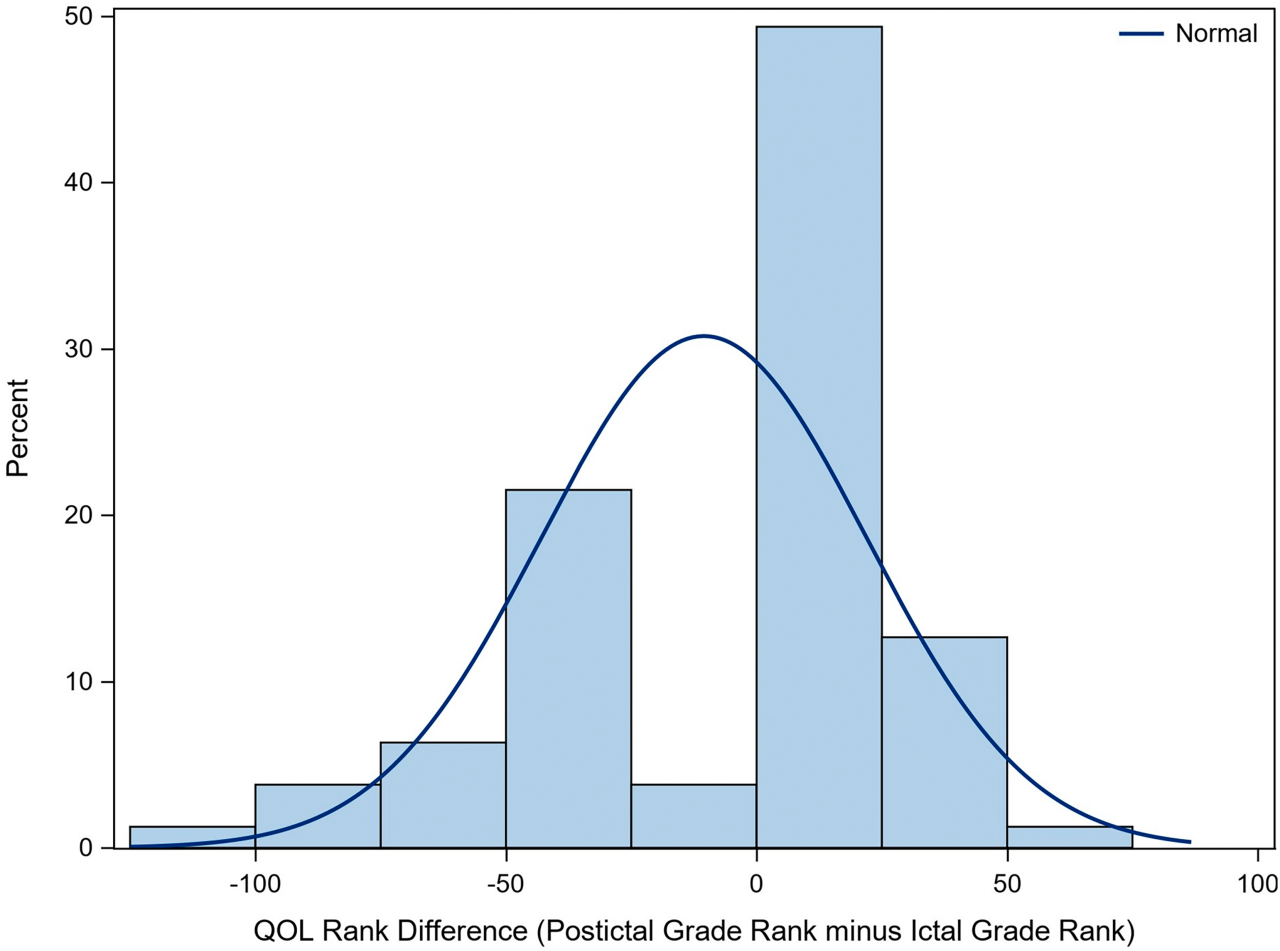
Quality of Life score rank difference plot showing the differences of rank between ictal scores and PI scores for each idiopathic epileptic dog, scored by the owner. The PI score was subtracted by the ictal score. The width of the bars of this histogram represents binned values in increments of 25 (eg, 0‐25). The percentage of observations equates to the bar height. The curve on the plot acts as a reference to reinforce the fact that the distribution is not normal.

The impact of PI signs on QOL was reported to be similar to the ictal phase in 37/79 (46.8%; 95% CI: 35.5%‐58.4%) dogs, greater than the ictal phase in 27/79 (34.2%; 95% CI: 23.8%‐45.7%) dogs and less than the ictal phase in 13/79 (16.5%; 95% CI: 9.1%‐26.5%) dogs. Kornbrot ranked difference test identified a significant difference between ictal and PI grades on QOL (*S* = −201, *P* < .01). Figure 6 shows that the difference between PI grade and ictal grade rank skews to the higher percentage of observations being >0; indicating that post ictal signs had a greater impact on perceived QOL compared with ictal signs.

Of the dogs with PI signs, 24/79 (30.3%; 95% CI: 20.5%‐41.8%) owners reported a change in their dog's behavior during the year before questionnaire completion, including change in mental status (15), abnormal social interaction (12), inability to perform tasks (8) excessive vocalization (5), inappropriate chewing (4), and change in sleep pattern (4).

## 5 DISCUSSION

Our study provides insight into the key features and clinical relevance of the PI phase in dogs diagnosed with a Tier II confidence level of idiopathic epilepsy. We found that the majority of dogs in our study cohort exhibited PI signs (90%). The most common PI clinical signs were disorientation and wobbliness with most of the signs having a duration lasting between 1 and 30 minutes. These findings are in agreement with another study.^13^ In the aforementioned study, compulsive pacing was also a commonly reported sign, but this was only seen in 10% of dogs in our study. We also identified PI signs not previously reported in dogs, such as deafness and weakness on 1 side, which are analogous to reported PI signs in humans of auditory disturbances and Todd's paresis or paralysis, respectively. Todd's paresis or paralysis is thought to be caused by transient hypoperfusion immediately after generalized or focal seizure activity,^14^ which has been identified in both preclinical^15^ and clinical^16^ models. It has been suggested that these hypoperfusive events, in addition to playing a transient role in PI signs, might also have a more sustained effect on interictal behavior and cognition. Behavioral changes were identified in our study. However, these might not be exclusively caused by PI signs, because anti‐seizure medication or the ictal activity could also play a role in the development of behavioral abnormalities.

The duration of the PI state differs with respect to clinical manifestation in humans, with electroencephalography (EEG) suppression and unresponsiveness lasting seconds to minutes, Todd's paresis, confusion, and memory impairments lasting hours, and psychiatric problems lasting days to weeks. We represented our duration data in a similar way, and some signs (when seen) had a predilection for certain duration (eg, 70% of dogs with blindness only lasting seconds to minutes). However, no clear duration of time for each PI clinical sign was seen in our cohort of dogs.

Dogs with idiopathic epilepsy manifesting solely with focal seizures still had PI signs, and surprisingly all dogs that had focal seizures had observed PI signs. In addition, we identified the presence of observed PI signs in the absence of an ictal presentation. We recognize that the ictal phase could have been missed in some of these dogs because additional information was not obtained. Both of these findings have not been reported before and emphasize the difficulty in identifying various presentations of seizures in dogs, especially with absent and subtle focal ictal forms. Thus, the PI phase might act as a “footprint” for a seizure event with no obvious ictal signs.

The number of anti‐seizure medications varied in our study from 0 to 5 medications at a time. The only significant association identified with respect to anti‐seizure medication was that the addition of benzodiazepines resulted in a prolonged duration of PI signs. This observation highlights an important obstacle in characterizing PI signs because certain medication‐related adverse effects can result in similar signs as those seen in the PI period. Because benzodiazepines are commonly used for at‐home emergency management of seizures, the interpretation of this significance should be viewed with caution until further characterization of the transient effects of benzodiazepines is carried out. All other seizure medications, when added, did not alter PI signs. This finding is similar to what has been identified in human medicine, where none of the common anti‐seizure drugs had a significant effect on PI severe hypoxia when compared with vehicle treatment.^17^

We also evaluated the co‐existence of clinical signs in the hope that a cluster of clinical signs might act as a signature of a type of PI manifestations. We assessed a 1‐to‐1 association analysis that identified some logical associations, such as aggression with fear, and disorientation with unsteadiness. Three clinical signs that shared a significant mutual association of presence were deafness, disorientation, and fear. This constellation of signs interestingly has been documented in humans who have been diagnosed with temporal lobe epilepsy,^17^, ^18^ with this area of the brain housing the auditory cortex as well as centers for emotion and cognition. In our study, ictal clinical signs did not show any true correlation with other causes for generalized or focal epilepsies. This observation could be a true finding or associated with small sample size. However, the presence of individual signs or clusters of signs might provide insight into the localization of particular epileptogenic foci.

Our findings not only showed that the PI phase alone has an impact on QOL, but also when compared with the ictal phase. This finding emphasizes this under‐recognized phase having, in some cases, a more perceived detrimental effect on the dog. Further work would be needed to understand why such was the case and to isolate which dogs are most at risk of developing severe PI signs. Such studies then might serve a stronger reason to explore ways of managing the seizure phase. Currently, in human medicine, therapeutic trials are being undertaken to determine the effects of certain medications that target inhibition of cyclooxygenase‐2 and T‐ and L‐type calcium channels,^15^ which play roles in limiting the hypoperfusive effects that are thought to be associated with the PI period.

Our study had some limitations. A questionnaire‐based approach allowed a large amount of information to be collected, but recall bias is inevitable, thus calling into question the reliability of some aspects of the answers. An attempt to limit agreement or acquiescence bias also was achieved by evenly distributing the number of questions, targeting the dogs' general seizure history, and not focusing on the PI phase alone. Also, the reliability of differentiating the various signs could be difficult (eg, differentiating between weakness and unsteadiness and wobbliness). Differentiation between when ictal signs stop and PI signs start has always been challenging.^19^ Electroencephalograpic seizure activity also has been shown to persist, although the ictal clinical signs improve.^20^ Therefore, to determine the true termination of the ictal phase, EEG would need to be utilized, requiring the study to be performed in a clinical setting. Information was obtained about the client‐owned dogs' seizure activity, with an attempt to differentiate among types of seizures, duration, repeatability, and whether other factors played a role. However, because every dog's ictal phase and PI phase of every individual seizure were not documented, links between the phases and semiology should be interpreted cautiously.

## 6 CONCLUSION

The PI phase of a seizure might be a more clinically relevant phase than previously thought, with our study findings suggesting that it not only might provide a footprint of the ictal phase but could also be a separate entity, resulting in changes in prognosis and effectiveness of the intervention. Further study in dogs is warranted into the prognostic and relevant value of the PI phase in dogs with idiopathic epilepsy.

## CONFLICT OF INTEREST DECLARATION

Authors declare no conflict of interest.

## OFF‐LABEL ANTIMICROBIAL DECLARATION

Authors declare no off‐label use of antimicrobials.

## INSTITUTIONAL ANIMAL CARE AND USE COMMITTEE (IACUC) OR OTHER APPROVAL DECLARATION

Authors declare no IACUC or other approval was needed.

## HUMAN ETHICS APPROVAL DECLARATION

Authors declare human ethics approval was not needed for this study.

## Supporting information


**Data S1.** Supporting Information.
