# Correction to “Characterization of Post‐Ictal Clinical Signs in Dogs With Idiopathic Epilepsy: A Questionnaire‐Based Study”

**DOI:** 10.1111/jvim.70265

**Published:** 2025-11-06

A. Nagendran, J. A. Nettifee, D. Carter, and K. R. Muñana, “Characterization of Post‐Ictal Clinical Signs in Dogs With Idiopathic Epilepsy: A Questionnaire‐Based Study,” *Journal of Veterinary Internal Medicine* 39, no. 1 (2025): e17302, https://doi.org10.1111/jvim.17302.

In the article cited above, minor discrepancies were found in the Abstract (“Results”) when compared to the Results in section 4.1 (“POST‐ICTAL SIGNS”).

The corrected sections are shown below; there is no impact on the conclusions of this study.

In the abstract, these sentences

Median duration of PI signs was 30 minutes (range, 5–4320 minutes). The most common PI signs reported were disorientation (50/79) and wobbliness or clumsiness (49/79).

Should be changed to

Median duration of PI signs was 30 minutes (range, **1–4320** minutes). The most common PI signs reported were disorientation (**71/79**) and wobbliness or clumsiness (**67/79**).

We apologize for this error.

