# Supplementary material for: Characterization of post‐ictal clinical signs in dogs with idiopathic epilepsy: A questionnaire‐based study
Source: J Vet Intern Med. 2025 Jan 20;39(1):e17302. doi: 10.1111/jvim.17302 (PMC11744365; doi:10.1111/jvim.17302)
Supplement: Supplementary file 1 — Data S1. Supporting Information. [file JVIM-39-e17302-s001.pdf]

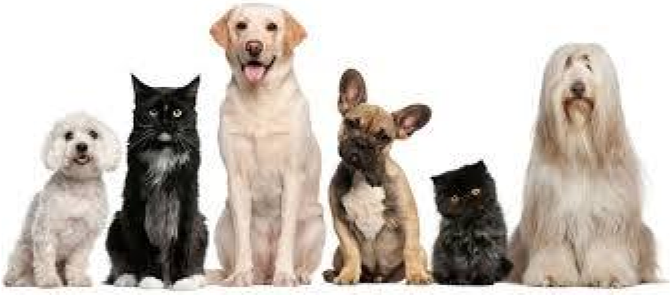

North Carolina State University
College of Veterinary Medicine
Epilepsy History Form

Main Survey

..
Thank you for participating in this survey. We, at NC State Neurology Service, are committed to furthering our understanding of canine epilepsy. With this questionnaire, we are hoping to gain more information about what happens to your dog immediately before, during and, in particular, immediately AFTER (post-ictal) having a seizure . If your dog does not experience any abnormalities immediately before or after a seizure, we would still recommend completing the survey. With your help, we hope to be able to characterize these signs and assess the impact they have on dogs with epilepsy.

Participation in this survey is voluntary.
All responses will be anonymized and
identifying information for you
or your pet will not be shared.

Q1. Owner's Last Name (We are asking for your last name to link any relevant medical records with your survey responses)

Q2. Dog's Name

Q3. Are there any dogs that are related to your dog that are known to have epilepsy?

- ○ Yes; Relationship status: ____
- ○ No
- ○ Unknown

Q4. If yes, please share your dog's pedigree if available:

..

The following questions will be focused on your dog's seizure history.

Q5. How old was your dog when he/she had the first seizure? (Please answer in months)

Q6. Provide the date of the first seizure, if known (mm/dd/yy):

Q7. How long has your dog been experiencing seizure activity? (Please answer in months)

Q8. What time of day do your dog's seizures tend to occur?

- ☐ Morning
- ☐ Afternoon
- ☐ Evening

- [ ] Night
- [ ] No specific time of day

Q9. Is your dog's behavior normal between seizures?

- ○ Yes
- ○ No

Q10. Under which of the following circumstances does your dog's seizure activity usually occur? (More than one answer can be given)

- [ ] At rest
- [ ] During sleep
- [ ] During exercise
- [ ] During stress/excitement
- [ ] Soon after a meal
- [ ] During a thunderstorm/fireworks

Q11. If not mentioned in the question previously, have you noticed any other triggers to your dog's seizures?

- ○ Yes (please describe)
- ○ No

Q12. On average, how often has your dog had a seizure episode in the last 12 months?

- ○ Daily
- ○ Weekly
- ○ Monthly
- ○ Every 2-6 months

- Yearly

Q13. Has your dog ever had a more than one seizure within 24 hours?

- Yes
- No

Q14. How often does your dog have a seizure more than once a day?

- With every seizure occurrence
- With some seizure occurrence

Q15. What is the average number of seizures your dog has had in one day?

- 2
- 3
- 4
- >4

Q16. How long ago was your dog's last seizure event? (Please answer in days, if you don't know the number days, please write the date)

Q17. In summary, which statement fits best with your dog's most common seizure presentation

- My dog has isolated seizures only (one a day at the very most)
- My dog has cluster seizures (more than one seizure a day)
- My dog has both isolated seizures and cluster seizures on separate occasions
- My dog has long seizures that last more than 5 minutes (status epilepticus)

..

In this section, we will be asking you questions concerning what happens immediately BEFORE the seizure event.

Q18. When your dog has a seizure, how often have you been able to tell in advance that this will happen?

- All the time
- Sometimes
- Never

Q19. If you can tell if your dog is going to have a seizure, how long before the seizure can you tell?

- Days
- Hours
- Minutes

Q20. My dog displays the following warning signs ***BEFORE*** having a seizure:

	Never	Sometimes	Always
Attention seeking	○	○	○
Fearful	○	○	○
Aggressive	○	○	○
Sleepy/lethargic	○	○	○
Wobbly/clumsy	○	○	○
Blind	○	○	○
Disoriented	○	○	○
Vocalization	○	○	○
Weak on all four legs	○	○	○
Wear on back legs only	○	○	○
Weak on one side (one front leg and one back leg)	○	○	○
Hungry	○	○	○
Thirsty	○	○	○

	Never	Sometimes	Always
Deafness	○	○	○
Other: Please describe:	○	○	○

. .

In this section we will be asking you questions about what happens DURING the seizure event

Q21. How long does one of your dog's seizure episodes typically last?

- ○ < 1 minute
- ○ 1-3 minutes
- ○ 3-5 minutes
- ○ > 5 minutes

Q22. Which of these following signs are seen **DURING** a seizure episode?

	Never	Sometimes	Always
Jaw chomping	○	○	○
Lip licking/ smacking	○	○	○
Biting the air/ " Fly biting"	○	○	○
Running movements of limbs	○	○	○
Stiffness of limbs	○	○	○
Loss of tone or floppiness of limbs	○	○	○
Vocalization	○	○	○
Rhythmic body or limb shaking	○	○	○
Increased drooling/ salivation	○	○	○
Urination	○	○	○
Defecation	○	○	○
Twitching of the face	○	○	○

	Never	Sometimes	Always
Other: Please describe: ☐	◯	◯	◯

Q22a. If running movements are seen, which side is usually affected?

- ◯ Right
- ◯ Left
- ◯ Both sides

Q22b. If stiffness is seen, which side is usually affected?

- ◯ Right
- ◯ Left
- ◯ Both sides

Q22c. If loss of tone or floppiness is seen, which side is usually affected?

- ◯ Right
- ◯ Left
- ◯ Both sides

Q22d. If rhythmic body or limb shaking is seen, which side is usually affected?

- ◯ Right
- ◯ Left
- ◯ Both sides

Q22e. If twitching is seen, which side is usually affected?

- ◯ Right
- ◯ Left
- ◯ Both sides

Q23. Do your dog’s seizures always look the same?

- ○ Yes
- ○ No

Q24. Which of the following best fits what you see in your dog **DURING** a seizure event?

	Never	Sometimes	Always
Whole body and head affected	○	○	○
Head affected only	○	○	○
Body affected only	○	○	○
Right side of the body affected only	○	○	○
Left side of the body affected only	○	○	○
Right side of the head affected only	○	○	○
Left side of the head affected only	○	○	○
No obvservable movement or twitches	○	○	○

Q25. What part of the body is usually involved first?

- ○ Head
- ○ Forelimbs
- ○ Hindlimbs

Q26. What is your dog's typical body position during a seizure?

- ○ Standing
- ○ Sitting
- ○ Laying on one side
- ○ Laying on stomach
- ○ Laying on back

Q27. If your dog lies on its side during the seizure, is it always the same side?

- ○ Yes
- ○ No
- ○ Not sure
- ○ Not applicable

Q28. Is your dog usually able to look you in the eyes or otherwise respond to you during the seizure?

- ○ Yes
- ○ No
- ○ Not sure

..

In this section, we will be asking questions concerning symptoms IMMEDIATELY AFTER your dog has a seizure event. We shall be calling these symptoms "after-seizure" signs

Q29. Does your dog exhibit any abnormal behavior immediately after a seizure occurs ("After-seizure" signs)?

- ○ Yes
- ○ No
- ○ Sometimes

Q30. How long after a seizure does it take for your dog to return back to normal?

- ○ Minutes (please specify) ______
- ○ Hours (please specify) ______
- ○ Days (please specify) ______

Q31. Which of the following abnormal signs does your dog display immediately **AFTER** having a seizure ("after-seizure signs"):

	Never	Sometimes	Always
Attention seeking	○	○	○
Fearful	○	○	○
Aggressive	○	○	○
Sleepy/lethargic	○	○	○
Wobbly/clumsy	○	○	○
Blind	○	○	○
Disoriented	○	○	○
Vocalization	○	○	○
Weak on all four legs	○	○	○
Weak on back legs only	○	○	○
Weak on one side (one front leg and one back leg)	○	○	○
Hungry	○	○	○
Thirsty	○	○	○
Deafness	○	○	○
Other: Please describe: ______	○	○	○

Q63. Which of the following abnormal signs does your dog display immediately **AFTER** having a seizure ("after-seizure signs"):

	Never	Sometimes	Always
Attention seeking	○	○	○
Fearful	○	○	○
Aggressive	○	○	○
Sleepy/lethargic	○	○	○
Wobbly/clumsy	○	○	○
Blind	○	○	○
Disoriented	○	○	○
Vocalization	○	○	○
Weak on all four legs	○	○	○

	Never	Sometimes	Always
Weak on back legs only	○	○	○
Weak on one side (one front leg and one back leg)	○	○	○
Hungry	○	○	○
Thirsty	○	○	○
Deafness	○	○	○
Other: Please describe:	○	○	○

Q31a. If one front leg and back leg were weak, which side was affected?

- ○ Right side
- ○ Left side

Q32. "After-seizure" signs can sometimes appear to be sequential (different signs appear at different time points). Have you noticed this in your dog?

- ○ Yes
- ○ No
- ○ Not sure

Q33. Which "after-seizure" signs appear and when and how long do they last following the seizure?

	Seconds to Minutes	Minutes to Hours	Hours to Days	Not Applicable
Attention seeking	○	○	○	○
Fearful	○	○	○	○
Aggressive	○	○	○	○
Sleepy/lethargic	○	○	○	○
Wobbly/ Clumsy	○	○	○	○
Blind	○	○	○	○
Disorientated	○	○	○	○
Vocalization	○	○	○	○

	Seconds to Minutes	Minutes to Hours	Hours to Days	Not Applicable
Weak on all four legs	○	○	○	○
Weak on back legs	○	○	○	○
Weak one side	○	○	○	○
Hungry	○	○	○	○
Thirst	○	○	○	○
Other ________	○	○	○	○

Q34. Do you notice the presence of "after-seizure" signs more commonly following any of these events below

- ☐ Short isolated seizure <2min
- ☐ Long isolated seizure >2 min
- ☐ Status epilepticus >5min
- ☐ Cluster seizure activity (>1 seizure within a day) of short seizures
- ☐ Cluster seizure activity (>1 seizure within a day) of long seizures

Q35. Have these "after-seizure signs" changed in **duration** now compared to when your dog started having seizures?

- ○ Increased in duration
- ○ Decreased in duration
- ○ Stayed the same

Q36. In the last year, how have the seizure and "after-seizure" signs changed?

	Increased	Decreased	Stayed the same
Seizure Duration	○	○	○
Seizure Frequency	○	○	○
"After seizure" signs duration	○	○	○

	Increased	Decreased	Stayed the same
"After seizure" signs severity	○	○	○

Q36a. Which statements best apply to the change in the number of seizures seen in your dog in the last year (more than one statement can be ticked)

- ☐ increase in number in the last year, no more than one isolated seizure a day
- ☐ increase in number from one isolated seizure a day to more than one seizure a day (cluster)
- ☐ increase in frequency of each cluster of seizures
- ☐ increase in number of seizures within a cluster event
- ☐ other ______

Q37. Have you noticed a change in the type of "after-seizure" signs since your dog started having seizures?

- ○ Yes
- ○ No

Q37a. If you have noticed a change in the type of "after-seizure" signs, please indicate any of the following that apply:

	AFTER-SEIZURE SIGNS	
	Present at INITAL ONSET of your dog's seizures	Present at the MOST RECENT seizure
Attention seeking	☐	☐
Fearful	☐	☐
Aggressive	☐	☐
Sleepy/lethargic	☐	☐
Wobbly/clumsy	☐	☐
Blind	☐	☐
Disoriented	☐	☐
Agitated	☐	☐
Vocalization	☐	☐

	AFTER-SEIZURE SIGNS	
	Present at INITAL ONSET of your dog's seizures	Present at the MOST RECENT seizure
Weak on all four legs	☐	☐
Weak on one side (one front leg and one back leg)	☐	☐
Deafness	☐	☐
Other (please describe) ________	☐	☐

Q38. Have you seen any of these "after-seizure" signs in the absence of a preceding seizure?

○ Yes

○ No

..

In this final section, you will be asked questions regarding your dog's treatment and your dog's quality of life.

Q39. Which of the following medications has been used to treat seizures in your dog? Include medications given in the past as well as those currently being administered.

☐ Phenobarbital

☐ Potassium Bromide

☐ Gabapentin

☐ Zonisamide

☐ Benzodiazepines (diazepam, midazolam or lorazepam)

☐ Levetiracetam/Keppra

☐ Other ________

Q40. Has the addition of any medication had an effect on the "after-seizure" signs?

○ Yes

○ No

Q41. Has the addition of any daily anti-seizure medication affected your dog's "after-seizure" signs (please tick the appropriate circle which best applies to your dog)

	"After-seizure" signs					
	Increase in duration	Decrease in duration	Increase in severity (more signs)	Decrease in severity (less signs)	No change seen	Not on medication
Phenobarbital	☐	☐	☐	☐	☐	☐
Zonisamide	☐	☐	☐	☐	☐	☐
Potassium Bromide	☐	☐	☐	☐	☐	☐
Levetiracetam/Keppra	☐	☐	☐	☐	☐	☐
Gabapentin	☐	☐	☐	☐	☐	☐
Pregabalin	☐	☐	☐	☐	☐	☐
Benzodiazepines (Diazepam, Midazolam Chlorazapate, Clonazapam)	☐	☐	☐	☐	☐	☐

Q42. Does your dog currently exhibit any of the following behaviors in between seizures as compared to before the seizures started? (select all that apply)

☐ Change in mental status (depressed, hyperactive)

☐ Unable to perform previously learned tasks

☐ Abnormal social interaction (clingy, hiding, etc)

☐ Aggression

☐ Abnormal mentation (staring into corners, pressing head into walls, pacing)

☐ Inappropriate chewing / destruction of property

☐ Excessive vocalization

☐ Change in sleep patterns

☐ Not applicable

Q43. How would you grade the impact of the seizure events on your dog's overall quality of life?

- 0-No impact
- 1-Very mild impact
- 2-Mild impact
- 3-Moderate impact
- 4-Significant impact
- 5-Severe impact

Q44. How would you grade the impact of the "after-seizure" signs specifically on your dog's overall quality of life?

- 0-No impact
- 1-Very mild impact
- 2-Mild impact
- 3-Moderate impact
- 4-Significant impact
- 5-Severe impact

..

The survey is complete. Thank you again for participating!

Addendum

Q1. What daily anti-seizure medication is your dog currently managed on?

- ☐ Phenobarbital
- ☐ Zonisamide
- ☐ Potassium Bromide
- ☐ Levetiracetam/Keppra
- ☐ Gabapentin

☐ Pregabalin

☐ Benzodiazepines (e.g Diazepam, Midazolam, Clonazapam, Chlorazepate)

Q64. What daily anti-seizure medication is your dog currently managed on?

☐ Phenobarbital

☐ Zonisamide

☐ Potassium Bromide

☐ Levetiracetam/Keppra

☐ Gabapentin

☐ Pregabalin

☐ Benzodiazepines (e.g Diazepam, Midazolam, Clonazapam, Chlorazepate)

Q2. Is your dog on any other medication that is NOT for his/her epilepsy

○ Yes

○ No

Q3. If yes, please list the current daily medication he/she receives

○ Click to write Choice 1

○ Click to write Choice 2

○ Click to write Choice 3

go.ncsu.edu/epilepsyresearch
